# Deciding for others as a neutral party recruits risk-neutral perspective-taking: Model-based behavioral and fMRI experiments

**DOI:** 10.1038/s41598-018-31308-6

**Published:** 2018-08-27

**Authors:** Akitoshi Ogawa, Atsushi Ueshima, Keigo Inukai, Tatsuya Kameda

**Affiliations:** 1 Department of Neurophysiology, Juntendo University School of Medicine, 2-1-1 Hongo, Bunkyo-ku, Tokyo, 113-8421 Japan; 2 Brain Science Institute, Tamagawa University, 6-1-1 Tamagawagakuen, Machida, Tokyo, 194-8610 Japan; 3 Labratory for Symbolic Cognitive Development, RIKEN Center for Biosystems Dynamics Research, 2-1 Hirosawa, Wako, Saitama, 351-0198 Japan; 4 Department of Social Psychology, The University of Tokyo, 7-3-1 Hongo, Bunkyo-ku, Tokyo, 113-0033 Japan; 5 Facutly of Economics, Meiji Gakuin University, 1-2-37, Shirokanedai, Minato-ku, Tokyo, 108-8636 Japan

## Abstract

Risky decision making for others is ubiquitous in our societies. Whereas financial decision making for oneself induces strong concern about the worst outcome (maximin concern) as well as the expected value, behavioral and neural characteristics of decision making for others are less well understood. We conducted behavioral and functional magnetic resonance imaging (fMRI) experiments to examine the neurocognitive underpinnings of risky decisions for an anonymous other, using decisions for self as a benchmark. We show that, although the maximin concern affected both types of decisions equally strongly, decision making for others recruited a more risk-neutral computational mechanism than decision making for self. Specifically, participants exhibited more balanced information search when choosing a risky option for others. Activity of right temporoparietal junction (rTPJ, associated with cognitive perspective taking) was parametrically modulated by options’ expected values in decisions for others, and by the minimum amounts in decisions for self. Furthermore, individual differences in self-reported empathic concern modified these attentional and neural processes. Overall, these results indicate that the typical maximin concern is attenuated in a risk-neutral direction in decisions for others as compared to self. We conjecture that, given others’ diverse preferences, deciding as a neutral party may cognitively recruit such risk-neutrality.

## Introduction

Humans often have to make decisions on behalf of others, particularly in situations involving financial and social services. Such decisions often require political considerations, as they involve a great deal of uncertainty about consequences that affect the lives of many others whom decision makers do not know personally. However, the neurocognitive bases of such decisions for others are not well understood.

It is well established that decision making for oneself deviates systematically from a simple maximization of expected value, due to the element of risk. Besides the expected value, people are concerned with how variable (variability concern^1,2^) or how bad (maximizing the minimum payoff, i.e., maximin concern^3,4^) choice outcomes can be, generally exhibiting risk-averse preferences. On the other hand, research suggests that risky decision making for others may be less affective and more risk tolerant than decision making for self ^5^. Hsee & Weber^6^ showed that people tend to think that others are less risk averse than themselves, whether the choices are between options with positive or negative outcomes. According to such predictions, people may focus more strongly on the expected values of choice options when they decide for others than for self. It is also conceivable that, given the relative lack of preference information for others compared to oneself, using the expected value as a normative criterion may be politically most justifiable in risky decision making for others.

Considering the importance of such a potential self-other difference, rigorous behavioral comparisons have been rather sparse (with a few notable exceptions^7–9^), not to mention research investigating its neural correlates. The difference could be reflected in multiple modalities during decision making, not only in choices *per se* but also in information search processes, personality traits, and brain activity. Using model-based behavioral and fMRI experiments, this study investigates how risky decisions for others compare to those for self in multiple modalities and how the possible self-other difference may be modulated by individual differences in empathy.

## Results

### Choice model

**Figure 1:**
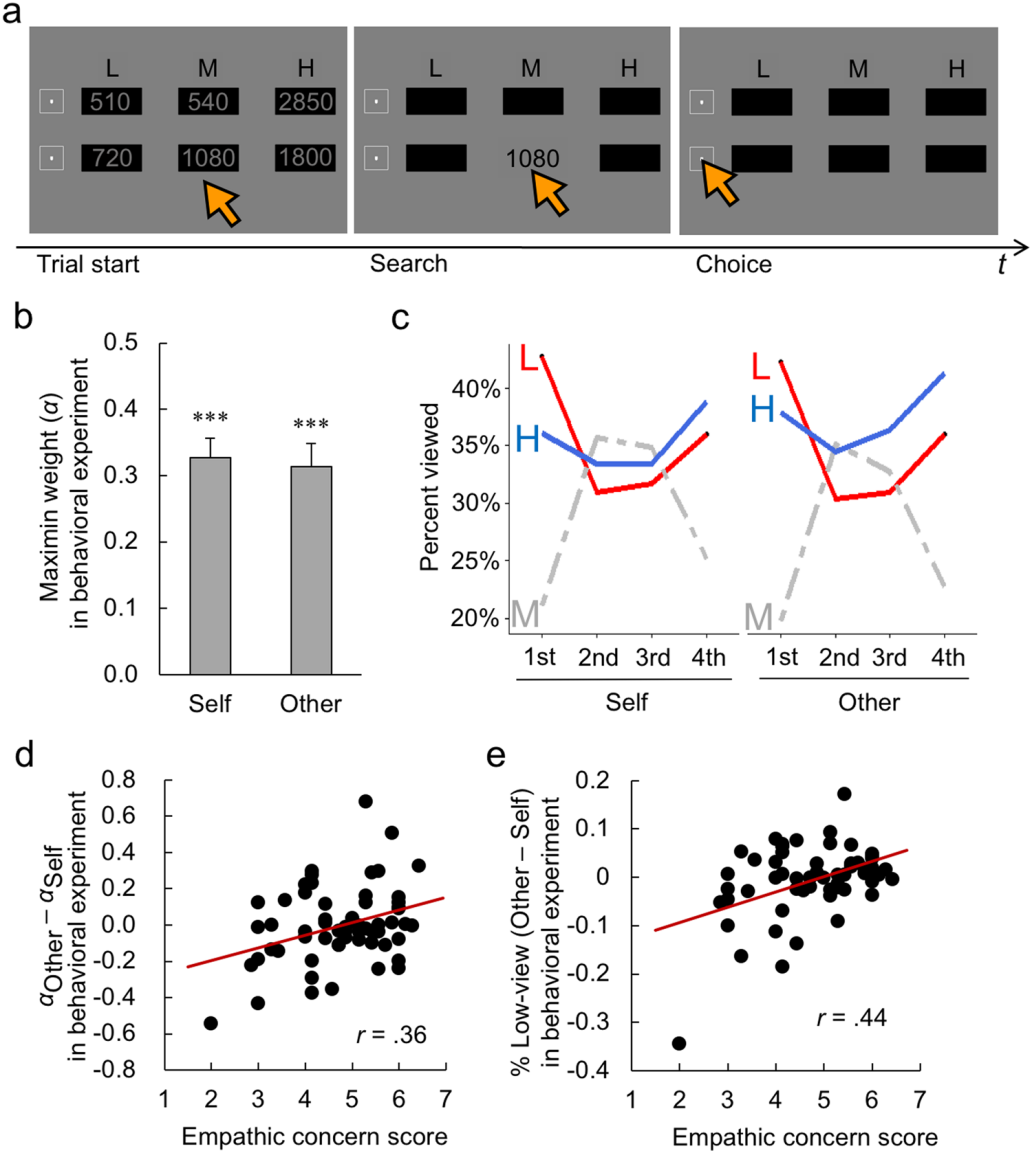
Stimulus and results from the behavioral (Mouselab) experiment. (**a**) A numerical example (in JPY) of the two choice options (left) and “Mouselab” interface (middle) displayed to participants. Numerical outcome information, initially hidden behind boxes labeled L, M, or H (low, medium, or high), was displayed only when the participant held the pointer over a box. A choice was confirmed by clicking the check mark to the left of the option within the 30 seconds of the trial. Column order was counterbalanced every 6 trials (LMH or HML). Row order of the two options was randomized across participants and choice pairs in each task. (**b**) Maximin weights *α*, estimated from participants’ choices in the Self and Other conditions. The asterisks indicate that both were significantly different from zero (***P < 0.001). (**c**) Temporal changes in the average percentages of L, M, or H views during the decision time used by each participant in each trial, divided into quartiles. (**d**) Significant correlation between participants’ self-reported empathic-concern scores and the difference between the maximin weights (*α*) in the Other and Self conditions. (**e**) Significant correlation between participants’ self-reported empathic-concern scores and the difference between the percentages of the low (“L”) box view in the Other and Self conditions.

In both the behavioral and fMRI experiments, participants made 36 risky choices each for self and for an anonymous other, with the task order counterbalanced. In each trial, they were asked to choose one of two lottery options consisting of three equiprobable outcomes (Fig. 1a left; Table S1). In the behavioral experiment (*N* = 60), the numerical outcome information, initially hidden behind boxes labeled L, M, or H (low, medium, or high), was displayed only while participants held the pointer over a box (the Mouselab technique^10^: Fig. 1a middle), allowing us to examine how participants attended to relevant information prior to making choices^3^.

For the analysis of behavioral choices, we adopted the following economic model (“quasi-maximin model”)^11^. Applied to the three-outcome (*π*_1_, *π*_2_, *π*_3_) cases, the model posits that the utility of option *x* for participant *i* is given by a linear combination of its minimum outcome and the expected value:

1 $${U}_{i}(x)={\alpha }_{i}\,{\rm{\min }}[{\pi }_{1},{\pi }_{2},{\pi }_{3}]+(1-{\alpha }_{i})\cdot EV,$$

where *α*_*i*_ ∈ [0, 1] captures the degree of individual concern about the minimum outcome (hereafter, the maximin weight). Setting *α* = 0 corresponds to completely risk-neutral preferences, in which the utility of an option is measured solely by its expected value, while setting *α* = 1 corresponds to completely maximin preferences where the option utility is determined solely by its minimum outcome. If *α*_*i*_ is between 0 and 1, individual *i* is concerned with the minimum outcome as well as the expected value. For each participant, we estimated the maximin weight for Self (*α*_self_) and Other (*α*_other_) conditions separately by fitting the 36 choices to the model using a logit analysis (see Table S2 for more details of model fitting).

### Behavioral experiment

The analysis revealed that the average maximin weights for Self and Other conditions were both significantly greater than zero in the behavioral experiment (Fig. 1b: one-sample *t* test, Self, *t*_59_ = 10.9, *P* < 0.001; Other, *t*_59_ = 8.94, *P* < 0.001). This means that most participants were risk averse, placing considerable weight on both the minimum outcome and the expected value in evaluating choice options, as suggested by previous research^3,4^. Participants also took more time to decide for Self than for Other (*M*_Self_ = 9.49 s, *M*_Other_ = 8.41 s; paired *t* test, *t*_59_ = 3.37, *P* < 0.01). However, no self-other difference in risk preference was detected at the decisional level: the maximin weights in choice preferences were not significantly different between the Self and Other conditions (*t*_59_ = 0.51, *P* = 0.61).

To examine information-search processes preceding decisions, we divided the decision time used by each participant in each trial into quartiles. As seen in Fig. 1c, participants tended to start information search by viewing the low (“L”) boxes in both conditions, prioritizing attention to the minimum outcomes in the early phase of decision making. Importantly, the degree of such selective attention to the worst outcomes (“L”) over the best outcomes (“H”) in the first view was significantly less evident in the Other condition than in the Self condition (generalized linear mixed model, *Z* = 2.85, *P* = 0.004). Furthermore, as the absolute difference in minimum outcomes between two choice options (ΔMin hereafter) was increased, participants viewed the larger-minimum option for longer than the smaller-minimum option in the Self condition (*P* = 0.008 by linear mixed model; see details in Methods), but not in the Other condition (*P* = 0.90). These results suggest that attentional bias toward the minimum may be relatively attenuated in risky decision making for others.

Attention to another’s potential misfortune may also be modulated by individual trait differences in empathic concern. Previous research has shown that likelihood of non-utilitarian decisions, which consider the welfare of the worst-off individual over the aggregated utility for the entire group in moral dilemmas, are positively correlated with empathic concern^12,13^, defined as “other-oriented feelings of sympathy and concern for unfortunate others”^14^. We measured participants’ empathic concern using the Interpersonal Reactivity Index (IRI)^14^. Figure 1d displays the relationship between this self-reported empathic concern and the difference between the maximin weights in the Other and Self conditions (*α*_Other_ − *α*_Self_). As seen in the figure, participants with higher empathic-concern scores tended to weight the worst possibility more heavily when making risky decisions for others, *r* = 0.36, *P* = 0.005 (Fig. 1d). Similarly, these more empathic participants also viewed the low (“L”) boxes more frequently during information search when deciding for others, *r* = 0.44, *P* < 0.001 (Fig. 1e). However, participants’ perspective-taking scores (defined as “the tendency to spontaneously adopt the psychological point of view of others” in the IRI^14^) revealed no similar patterns (Fig. S1). These results may imply that, even though decision making for others generally reduces cognitive focus on the minimums at the group level (Fig. 1c), individual trait differences in affective empathy may modulate the effect.

### fMRI experiment

**Figure 2:**
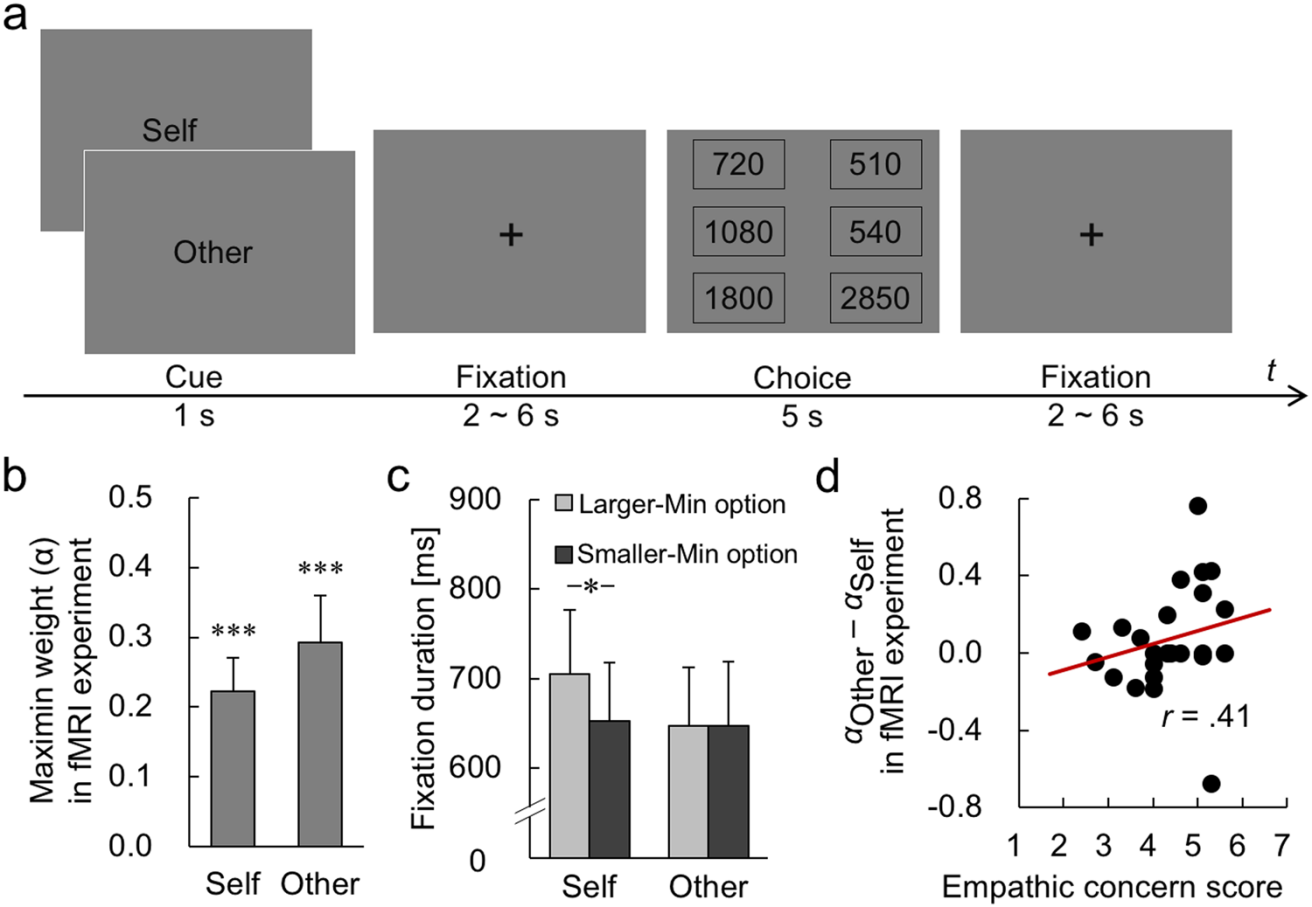
Task sequence and behavioral results from the fMRI experiment. (**a**) Task sequence. A cue indicating the condition of the current trial was presented for 1 s, followed by a fixation duration between 2 and 6 s (mean = 4 s). Two choice options were then presented vertically on the left and the right sides for 5 s. Inter-trial interval was between 2 and 6 s (mean = 4 s). The numeric structure of the options and the instructions were all identical to those in the behavioral experiment. (**b**) The maximin weights (*α*) estimated from participants’ choices in the Self and Other conditions. The asterisks indicate that both were significantly different from zero (***P < 0.001). (**c**) Eye tracking results. Because the size of the MRI monitor was not large enough to adequately separate the individual monetary outcomes, gaze durations on the two options were analyzed here. In the Self condition, participants looked more at the option with larger minimum amount (*P < 0.05), but such attentional bias was not observed in the Other condition. (**d**) Correlation between the participants’ self-reported empathic-concern scores and the difference between the maximin weights (*α*) in the Self and Other conditions.

To understand the neural correlates of such self-other differences, we conducted an fMRI experiment (*N* = 24). The task structure was identical to that used in the behavioral experiment, except that we measured participants’ information search behavior using an eye tracker instead of the Mouselab technique (Fig. 2a).

We first confirmed the behavioral and attentional results above. At the group level, participants exhibited substantive maximin concern in both Self and Other conditions (Fig. 2b: one-sample *t* test, Self, *t*_23_ = 4.64, *P* < 0.001; Other, *t*_23_ = 4.29, *P* < 0.001), but the self-other difference was not significant (*t*_23_ = 1.23, *P* = 0.23). Participants also took more time to make decisions for Self than for Other (*M*_Self_ = 2.59 s, *M*_Other_ = 2.51 s; paired *t* test, *t*_23_ = 2.39, *P* < 0.05). As in the behavioral experiment, participants exhibited biased attention to minimum outcomes, viewing the option with the larger minimum more in the Self condition (Fig. 2c: *t*_16_ = 2.41, *P* < 0.05), but this bias was not observed in the Other condition (*t*_16_ = 0.23, *P* = 0.82). Also, parametric differences in the minimum outcomes between two options (ΔMin) significantly predicted longer gaze on the larger-minimum option in the Self condition (*P* = 0.036 by linear mixed model) but not in the Other condition (*P* = 0.55). At the individual level, participants with higher empathic-concern scores also weighted the worst possibility more in decisions for others (Fig. 2d: robust correlation, *r* = 0.41, *P* < 0.05; to avoid potential undue influence of outliers in our small sample, we adopted robust correlation analysis^15,16^ throughout the fMRI experiment using the WRS2 package^17^ for R software^18^).

#### Brain activity

Having replicated the behavioral and attentional results, we then examined neural responses. The brain regions of interest were right temporoparietal junction (rTPJ; involved in perspective taking^19,20^), medial orbitofrontal cortex (mOFC; associated with affective processes during decision making^21,22^), and ventral striatum (VS; involved in computing utility of choice options^23^). Although it was not the main concern of this study, we also summarized whole-brain activation in Table S3.

Our recent fMRI study showed that the maximin concern when choosing between risky options for oneself (comparable to the Self condition here) was selectively tracked by neural activity in the rTPJ^3^. As rTPJ is involved in cognitively prospecting future conditions for oneself (“mental time travel”)^24,25^, we interpreted these results as participants taking the perspectives of their “future selves” in the worst-possible scenario (i.e., imagining “what if my choice results in the worst outcome?”)^3,26^. Here we tested whether participants’ cognitive perspective taking may operate in a more risk-neutral manner when they make decisions for others.

**Figure 3:**
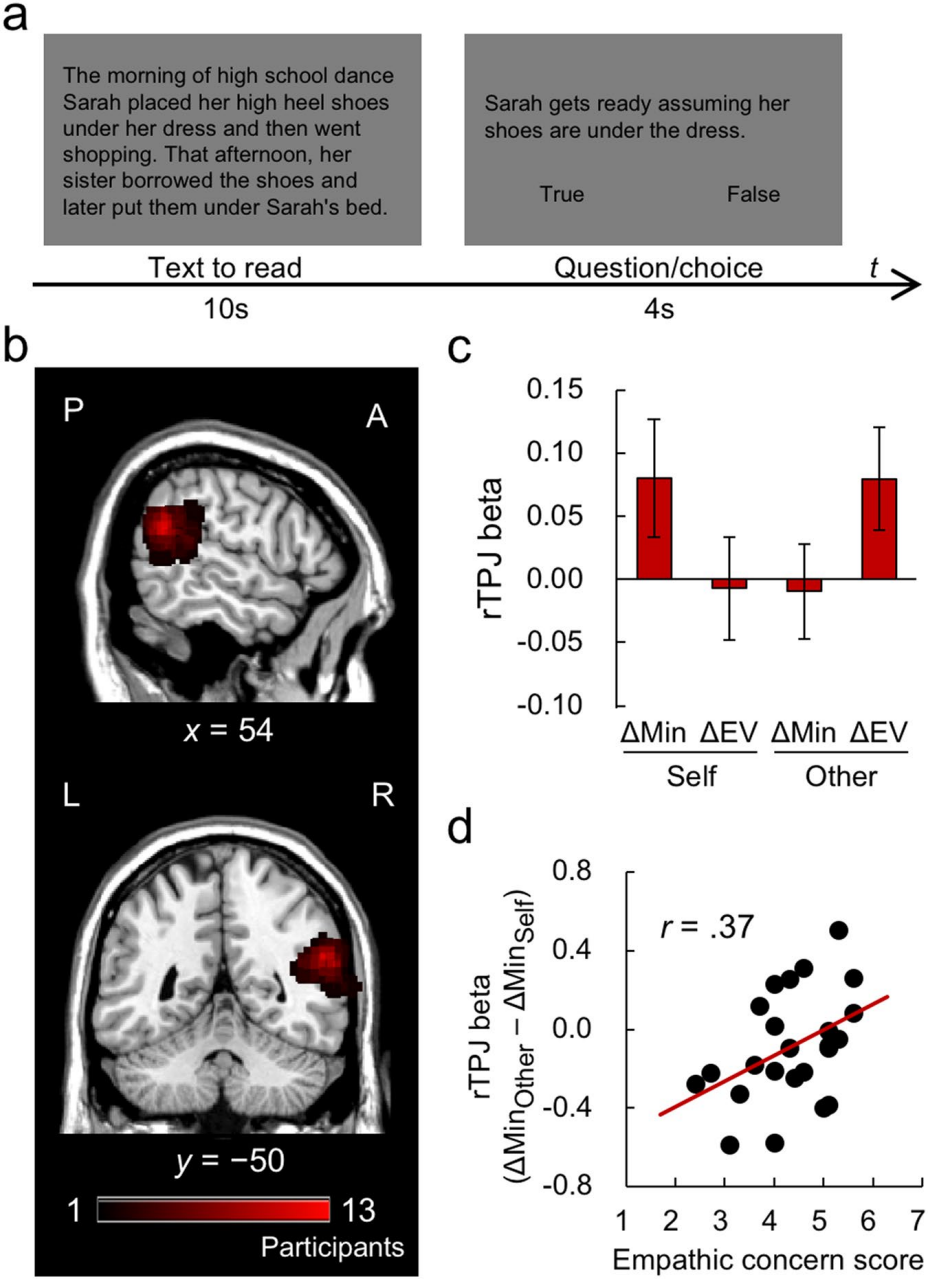
Imaging results regarding rTPJ from the fMRI experiment. (**a**) An example of using the Theory-of-Mind localizer to individually identify rTPJ ROI associated with cognitive perspective taking^27^. (**b**) Definition of individual rTPJ ROI on the sagittal section and the coronal section. The color bar shows the number of overlapped individual ROIs. L = left, R = right, A = anterior, P = posterior. (**c**) Parametric modulation for ΔMin and ΔEV in the rTPJ activity. The interaction of the modulatory effect (ΔMin/ΔEV) and the task condition (Self/Other) was significant. (**d**) Significant correlation between participants’ self-reported empathic-concern scores and the difference between the modulatory effects of ΔMin on the rTPJ activity in the Other and Self conditions.

Using a Theory-of-Mind localizer^27^ (Fig. 3a) in a separate fMRI scan after the main experiment, we identified the rTPJ region correlated with cognitive perspective taking for each participant individually (see ROI analysis in Methods). It is important to note that, in contrast to the main task, the Theory-of-Mind localizer involved no elements of money or risk. The average coordinates of identified rTPJ regions were situated in the angular gyrus, corresponding to the canonical “social TPJ”^19^ (Fig. 3b). We examined whether activity in the identified rTPJ during decision making was modulated by the key parameters of the behavioral-choice model (Eq. 1) – the absolute difference in minimum (ΔMin) and absolute difference in expected value (ΔEV) between the two options. If a participant’s cognitive perspective taking centers on the worst-possible scenario (or the expected value), it is expected that their rTPJ activity will track how the two options compare in terms of minimum outcomes (or the expected values). Figure 3c displays the modulatory effects of the two parameters (ΔMin and ΔEV) on rTPJ activity in the Self and Other conditions. A two-way repeated measures ANOVA with condition (Self or Other) and parameter (ΔMin or ΔEV) revealed a significant interaction, *F*_2,46_ = 4.82, *P* = 0.038. This implies that cognitive perspective taking operated on different dimensions in the Self and Other conditions; specifically, the former centered on the worst-possible outcome (ΔMin) in line with our recent fMRI study^3^ whereas the latter focused on the risk-neutral, average outcome (ΔEV).

We also examined whether individual differences in empathic concern may modulate cognitive perspective taking of another’s misfortune. Figure 3d shows the relation between self-reported empathic concern and the modulation of rTPJ activity by ΔMin in the Other condition. Although the effect was weak, participants with higher empathic concern exhibited greater rTPJ activity in response to ΔMin (robust correlation, *r* = 0.37, *P* = 0.081) in line with the behavioral results (Figs 1d,e and 2d; see also Fig. 1). This may indicate that, even though decision making for others generally recruits cognitive perspective taking via ΔEV (rather than ΔMin) at the group level, individual trait differences in empathic concern also modulate the effect.

**Figure 4:**
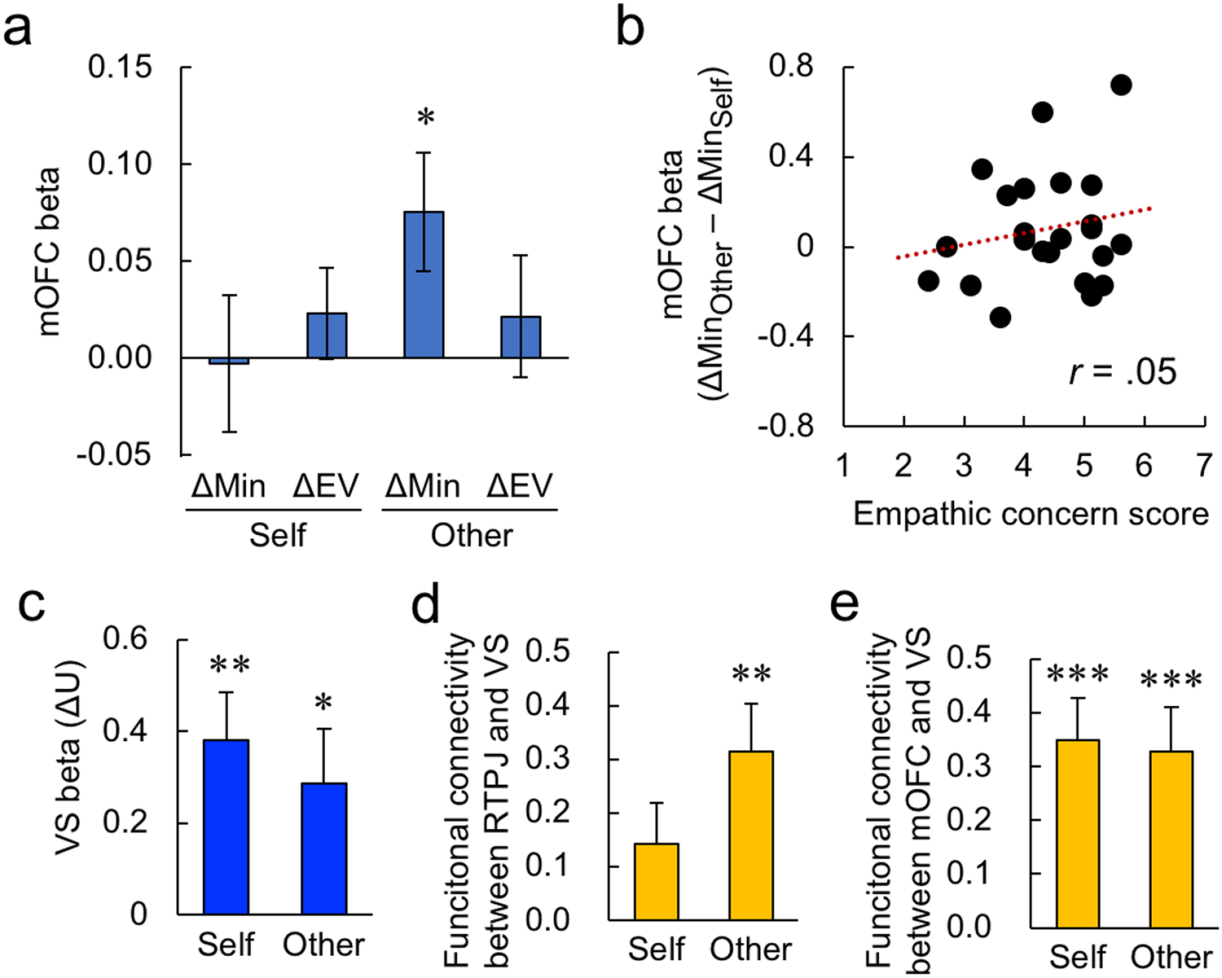
Imaging results regarding mOFC, VS, and functional connectivity from the fMRI experiment. (**a**) Parametric modulation for ΔMin and ΔEV in the mOFC activity. The asterisk indicates a significant difference (*P < 0.05). (**b**) Non-significant correlation between the participants’ self-reported empathic-concern scores and the difference between the modulatory effects of ΔMin on the mOFC activity in the Other and Self conditions. (**c**) Parametric modulation for ΔU in the VS activity during decision-making (**P < 0.01, *P < 0.05). (**d**) Functional connectivity between rTPJ (seed) and VS (target) during decision-making (**P < 0.01). (**e**) Functional connectivity between mOFC (seed) and VS (target) during decision-making (***P < 0.001).

Besides cognitive processes, affective processes may also be evoked when participants make risky decisions on behalf of others. The medial orbitofrontal cortex (mOFC) is associated with emotive non-utilitarian decisions for others in moral dilemmas (i.e., sympathizing with the worst-off individual^28^) and guilty feelings about another’s disadvantaged payoff in economic games^29^. We thus examined whether activity in the mOFC (see ROI analysis in Methods) was modulated by the two task parameters of the choice model. As seen in Fig. 4a, activity in mOFC tracked ΔMin in the Other condition (*t*_23_ = 2.48, *P* < 0.05 after Bonferroni family-wise error correction). However, mOFC activity in response to ΔMin was not modulated by individual differences in empathic concern (Fig. 4b: robust correlation, *r* = 0.05, *P* = 0.80), suggesting that the emotive reaction to another’s (possible) misfortune was relatively common across participants. These results imply that, besides the cognitive perspective taking along the risk-neutral expected value outcome (Fig. 3c), some emotive process is also evoked by the worst-off possibility, affecting risky decision making for others at the group level.

The economic choice-model (Eq. 1) suggests that such neural activities should be integrated into calculations of option utilities. We first confirmed that the ventral striatum (VS), which is associated with comparison of option utilities^23^, was involved in this process. Using the average maximin weights of all participants (mean *α*_self_ and mean *α*_other_), we calculated the absolute differences in utility between the two options (ΔU) for each choice according to the model. We then examined whether activity in the VS (see ROI analysis in Methods and Fig. S2) was parametrically modulated by ΔU, separately in the Self and Other conditions. As shown in Fig. 4c, the modulatory effect was significant in both conditions (Self, *t*_23_ = 3.62, *P* < 0.01; Other, *t*_23_ = 2.37, *P* < 0.05: both *P*’s after Bonferroni correction), confirming that the VS encoded the utility difference between two options.

#### Functional connectivity

We next examined whether the VS was functionally connected with the rTPJ and mOFC, which tracked the key parameters of the choice model (Figs 3c and 4a). We performed a psychophysiological interaction (PPI) analysis with seeds of rTPJ and mOFC and the target ROI of VS during decision-making. The beta estimate of the PPI was weak in the Self condition but significant in the Other condition (Fig. 4d for rTPJ: Self, *t*_23_ = 1.84, *P* = 0.16; Other, *t*_23_ = 3.43, *P* < 0.01; Fig. 4e for mOFC: Self, *t*_23_ = 4.50, *P* < 0.001; Other, *t*_23_ = 3.99, *P* < 0.001: all *P*’s after Bonferroni correction), suggesting that both cognitive perspective-taking (rTPJ) and affective/emotive processes (mOFC) were involved in utility calculations in risky decision making for others.

## Discussion

This study investigated multimodal processes underlying risky decision making for others. Although deciding matters on behalf of others is common across many financial, social, and political situations, the neurocognitive underpinnings of such decisions are not well understood. Here we have addressed this question by setting risky decisions for self as a benchmark for comparison^3^. The self-other comparison has revealed a systematic pattern across multiple modalities.

First, in line with previous studies^3,4^, participants showed a strong maximin concern across the behavioral and fMRI experiments, weighting the worst (minimum) possibility in risky decision making for others as well as for self (Figs 1b and 2b). Although the operation of maximin concern at the decisional level was indistinguishable between the Self and Other conditions, decision making for others recruited a more risk-neutral computational mechanism than decision making for self. Specifically, information search in the behavioral experiment (Fig. 1c) and gaze pattern in the fMRI experiment (Fig. 2c) both showed that attentional bias to the worst outcomes was attenuated in the Other condition compared to the Self condition. The brain-imaging results also showed that rTPJ activity was parametrically modulated by the difference in minimums (ΔMin) in the Self but not in the Other condition. Instead, rTPJ activity in the Other condition was modulated by the difference in expected value (ΔEV; Fig. 3c). It should be emphasized that the Theory-of-Mind localizer that we used to identify ROI of rTPJ for each participant (Fig. 3a) was purely cognitive, with no affective, risk, or monetary components. Taken together, these results imply that participants’ cognitive perspective taking operated in a more risk-neutral manner when deciding on behalf of others, mainly occurring along the dimension of expected value.

Simultaneously, the activity of mOFC, which relates to affective, non-utilitarian decisions for others^28,29^, was modulated by the worst possible outcome (ΔMin) when deciding for others (Fig. 4a). This suggests that, when deciding on behalf of others, people are emotionally concerned with the worst-off possibility, while cognitively focusing on the risk-neutral average scenario (ΔEV) as a neutral third party. The PPI results confirmed that both the cognitive and affective elements were integrated in utility calculations for risky decision making for others at VS (Fig. 4d,e), which may explain why the maximin concern was indistinguishable at the decisional level between the Self and Other conditions (Figs 1b and 2b).

Finally, this study revealed robust individual differences associated with empathy across multiple modalities. More empathic participants displayed greater maximin concern for others at the decisional level (Figs 1d and 2d), and this pattern was also observed at the attentional (Figs 1e and 2c) and neural (Fig. 3d) levels. These results prompt the question of whether these highly empathic participants may be particularly sensitive to potential harm to others, as suggested by previous research on neural responses to others’ pain^30,31^. Future neurocognitive research addressing the modulatory role of empathy in calculating values for self and for others is highly important, as empathy constitutes a core dimension of human sociality^32^.

By using multimodal measurements, we have shown that risky decision making for others engages more risk-neutral cognitive processes as compared to that for self. Whereas the maximin concern (which is likely to be an evolutionary adaptation for survival^3,33^) also affected final decisions for others through affective routes, its cognitive operation during decision making was attenuated. Given the diverse preferences of others, perhaps using expected value as a normative criterion for decision making would be politically most justifiable. In other words, deliberating about decisions for others as a “neutral third party” may entail cognitive risk neutrality; this would seem to have important implications for many financial and welfare decisions in our societies.

## Methods

### Overview

This study consists of two experiments. The first is a behavioral experiment using the Mouselab technique^10^ to investigate information search during decision making. The second is an fMRI experiment to reveal neural correlates of risky decisions for others as compared to those for self. In the fMRI experiment, participants’ eye movements during decision-making were recorded by an eye-tracker to examine whether the information search process was consistent with that identified by the Mouselab technique in the behavioral experiment. At the end of each experiment, each participant’s empathic concern was measured using a seven-point Likert-scale questionnaire (Davis’s Interpersonal Reactivity Index: IRI)^14^. Empathic concern is one of the subscales of IRI, assessing “other-oriented” feelings of sympathy and concern for unfortunate others^14^. After the experiment, participants received the base payment plus the result of a lottery, randomly drawn from their 36 choices for self. Both experiments were approved by and carried out in accordance with the guidelines and regulations of the ethical committee of the Center for Experimental Social Sciences of Hokkaido University.

### Participants

Sixty Hokkaido University students participated in the behavioral (Mouselab) experiment (34 males and 26 females; age 18–21, mean = 19.2 years, s.d. = 0.80). In the fMRI experiment, we scanned 24 right-handed Hokkaido university students with no history of neurological or psychiatric illness (14 males and 10 females; aged 18–21, mean = 19.2 years, s.d. = 1.06) and no overlap with participants in the behavioral experiment. All participants had normal or corrected-to-normal visual acuity. All participants gave written informed consent, approved by the ethical committee, prior to the experiments.

### Tasks

#### Behavioral (Mouselab) Experiment

Participants performed 36 choice-trials in both Self and Other conditions, 72 trials in total. In each trial, two options were presented horizontally on the upper and lower positions of the display (Fig. 1a left). Each option included three equiprobable outcomes (High, Middle, and Low). Spatial ordering of the three outcome amounts was alternated (High/Middle/Low or Low/Middle/High) every 6 trials. On the screen, numerical outcome information was hidden behind boxes labeled L, M, or H (Fig. 1a middle). When the mouse pointer was held over a box, its numerical information was displayed, and when the pointer was moved away, the information was hidden again, so that participants could only view one box at a time. Participants had to make choices within 30 seconds in each trial, during which they were free to view any boxes in any order. Participants were asked to choose one option by clicking the check mark to the left of the option (Fig. 1a right). In the Other condition, participants were instructed to make choices for an anonymous student who would receive the monetary outcome from the chosen lottery as a compensation for participating in another experiment.

We used a laptop computer with a 15.6-inch display (PC-GN256FSG8, NEC co., Japan) and a set of PsychoPy^34^ scripts to present stimuli and record responses. As compensation for participating in this experiment, participants received the result of a lottery, randomly selected from the 36 choices in the Self condition, in addition to a show-up fee of 200 JPY in cash. Participants were instructed that if they made no choice within the trial’s time limit, a penalty of 50 JPY would be imposed each time; however, this occurred only once throughout the behavioral experiment.

#### fMRI experiment

Participants performed the same lottery choice task as in the behavioral experiment (Fig. 2a). The instructions for the Self and Other conditions were identical to those used in the behavioral experiment, except that participants were also asked to choose the option with larger arithmetic average in separate filler trials (Comp condition). There were 36 trials for each of the three conditions, 108 trials in total. The conditions alternated every 12 trials in an order that was counterbalanced across participants.

In each trial, a condition cue was presented for 1 s, followed by a stimulus onset asynchrony of 2, 4, or 6 s (Fig. 2a) with the fixation cross. Then, two options were presented vertically on the left and right side of the fixation cross at the center of the display. Each lottery option had three equiprobable outcomes. Spatial ordering of the three outcome amounts (High/Middle/Low or Low/Middle/High, listed top to bottom) was alternated every 6 trials. Participants were asked to choose one of the two options by pressing a corresponding button within 5 s. The options were presented on the screen for the entire 5 s regardless of when the participant responded. Inter-trial interval was 2, 4, or, 6 s with a fixation cross.

After the lottery-choice task, participants were also scanned while performing a Theory-of-Mind task (Fig. 3a) to identify the right temporoparietal junction (rTPJ) associated with perspective taking^27^. Participants were asked to infer the beliefs of others (Belief condition) or the states of places and physical objects (e.g., a fountain) as a control (Photo condition). There were 10 trials in each condition. In each trial, the background explanation was presented for 10 s, followed by the statement related to the inference. Participants were asked to answer whether the statement was true or false by pressing a corresponding button within 4 s, during which time the question remained on the screen. Then the fixation cross was presented for 12 s until the next trial began.

Stimuli were presented on an MRI-compatible 32-inch LCD display (NordicNeuroLab, Norway) placed at back of the MR bore. An MRI-compatible response pad (Current Designs, USA) was used to record responses. To examine each participant’s information search process, we also recorded their eye movements during each trial (see the Eye-tracking in fMRI section below). We used a PsychoPy^34^ script to present stimuli and record responses and eye-tracking data during the fMRI experiment. Participants received a fixed compensation of 4,000 JPY plus the result of a lottery, randomly selected from the 36 choices in the Self condition, all in cash.

During the fMRI experiment, eye movements were recorded using an MRI-compatible eye-tracker operating at 500 Hz (EyeLink CL, SR Research Ltd.). Before scanning, calibration was performed using a sequence of 9-point fixations. To classify saccades and fixations in each trial, we used the following parameters: saccade velocity threshold >30°/s, saccade acceleration threshold >9500°/s^2^, and fixation duration threshold >100 ms. Fixations and saccades were correctly measured in 17 out of 24 participants; the remaining 7 participants were excluded from this analysis because of technical problems (e.g. reflection from eyeglasses or contact lenses, partial occlusion of pupil by eyelashes).

### Computational modeling of choice behavior

As described in the main text, our previous study showed that the “quasi-maximin model”^11^ (Eq. 1) provided the best fit to the lottery choices for self^3^. The model posits the utility function as a weighted combination of the minimum amount and the expected value of the option. The choice probability was modeled using a softmax function of the option utilities. We used the Broyden-Fletcher-Goldfarb-Shanno method for numerical estimation of the maximin weight *α* for each participant in each task, using R software^18^ for the behavioral experiment and Matlab software (www.mathworks.com) for the fMRI experiment.

### Analysis of information search in the behavioral experiment

We analyzed the information search process using the percentages of viewed lottery outcome amounts in each quartile of the decision time used by each participant in each trial. Based on our previous research^3^, we hypothesized that participants would show greater attention to the minimum amounts during the early phase of decision making. Therefore, the percentage of viewing low amounts (“L” in Fig. 1a) first was compared with that of the high amounts (“H”) using a generalized linear mixed model with the logit link (first views on the middle amounts [“M”] were excluded here, because the middle amounts were generally viewed much less often in both conditions; see Fig. 1c). We also analyzed differences between viewing durations of the larger- and smaller-minimum options using a linear mixed model, with the absolute differences in the minimum amounts (ΔMin) and in the average amounts (ΔEV) between the two options in each pair as independent variables. In both analyses, participants were treated as random effects, and the display order of the amounts (L/M/H or H/M/L from left to right: Fig. 1a) was also modeled as a dummy variable in the model. For these analyses, we used the lme4 package^35^ for R software^18^.

### Analysis of eye movements in the fMRI experiment

Analyzing gaze on individual monetary amounts was not possible, because the size of the monitor in the MRI did not allow us to display the amounts with adequate spatial separation for such an analysis. Instead, we analyzed participants’ gaze duration on the two presented lottery options, assuming that biased attention to the minimum outcomes would result in longer view duration on the option with larger minimum amount. Gaze durations from the option display onset to the response were examined in the Self and Other conditions (Fig. 2c). We further investigated which task parameter of the choice set affected the longer gaze on the option with larger minimum. To do this, we used a linear mixed model of differences in gaze duration between the larger- and smaller-minimum options, with the parametric differences in the minimum amounts (ΔMin) and in the average amounts (ΔEV) of the two options as the independent variables. The participants were treated as random effects, and the display order of the amounts (L/M/H or H/M/L from the top to the bottom: Fig. 2a) was also modeled as a dummy variable. For this analysis, we used the Matlab Statistics and Machine Learning Toolbox (www.mathworks.com/products/statistics).

### Image acquisition of fMRI

A 3T MR scanner (Prisma; Siemens Medical Systems, Erlangen, Germany) equipped for echo-planar imaging (EPI) was used to acquire functional magnetic resonance images. A 64-channel head-neck coil was used for radio frequency signal reception. Mild cushioning minimized participant’s head movement. Thirty-eight slices of functional images were acquired using blood oxygenation level dependent imaging (192 mm × 192 mm × 142.5 mm, in-plane resolution = 64 × 64, pixel size = 3 mm × 3 mm, thickness = 3 mm, 25% distance factor, TR = 2.0 s, TE = 25 ms). The slices were rotated 30° from the AC-PC line to the forehead to minimize artifacts due to the air in the sinus. The images covered the entire cerebrum after the rotation. We acquired 255 scans in each fMRI run of the lottery choice task and 260 scans in the functional localizer for rTPJ (see below). The first three scans in each fMRI run were discarded to ensure magnetization equilibrium.

### Image analyses

#### Pre-processing

We used SPM8 (Wellcome Department of Cognitive Neurology, University College London) working on Matlab to process the scanned images. We performed slice-timing correction using the middle slice as a reference, scan-to-scan realignment, normalization to the EPI template of SPM, resampling the images with a voxel size of 3 mm × 3 mm × 3 mm, and spatial smoothing (FWHM of Gaussian kernel = 8 mm isotropic). A high-pass filter of 128 s was used to remove low frequency noise in the main and control experiments.

#### General linear model analysis

The first-level analysis using a general linear model (GLM) included the three choice conditions (i.e. Self, Other, and Comp), the condition of task cue, and the condition of response. The onsets of choice condition were the presentation of options. The duration of these conditions was measured as the response time in each trial. The onset of the task cue condition was the appearance of the cue, and its duration was 1 s. All three task cues were collapsed into one condition. Responses were also collapsed into a single condition with duration zero. The blood oxygen level-dependent (BOLD) signal was modeled using the canonical hemodynamic response function included in the SPM software. Realignment parameters reflecting head motion estimated in preprocessing were used as nuisance regressors. In addition to these effects on the brain activation, we examined the modulatory effects of ΔU (GLM1) and of ΔMin and ΔEV (GLM2) in choice period.

#### ROI analysis

Using a separate fMRI scan after the main experiment with the Theory-of-Mind localizer^27^, we individually identified the rTPJ, associated with taking another’s perspective^19,20,27^. The average coordinates of the peak of identified rTPJ (57, −50, 20) were located in the posterior subdivision of rTPJ. This is consistent with results from a previous study indicating that the posterior rTPJ is involved in cognitive perspective-taking, using anatomical and functional connectivity^36^. We examined the modulatory effect of the key parameters in the quasi-maximin model (i.e. ΔMin and ΔEV) on the activation of rTPJ, as well as whether the individual variability in empathic personality trait influenced the effect of parametric modulation.

We hypothesized that the mOFC would be associated with affective/emotive evaluation of choice options. We used the anatomical mOFC ROI defined in AAL2^37^ for analysis. Beta estimates in this ROI were extracted and examined. We also hypothesized that the activation in VS would be associated with utility calculations (ΔU) of the quasi-maximin model. For this purpose, an anatomical ROI of VS was defined using MarsBaR^38^. The striatum ROI of the automated anatomical labeling equipped in MarsBaR within the range from −12 to 0 of MNI *z* coordinate of caudate ROI was used as the VS (Fig. S1). To calculate the option utility based on the quasi-maximin model (Eq. 1) for this analysis, we used the maximin weight (*α*) averaged across all participants in each condition.

#### Functional connectivity analysis

We hypothesized that the VS would be functionally connected with the rTPJ and mOFC. First, we examined the functional connectivity between the rTPJ and the VS using a generalized psychophysiological interaction (gPPI)^39^. The time series of brain activity in the rTPJ was extracted, and the interactions between the first Eigenvariate of extracted time series and task conditions (Self/Other) were entered into a GLM analysis for gPPI. The beta estimates (i.e. functional connectivity) of the interactions in Self and Other conditions in the ROI of VS were extracted using MarsBaR and tested statistically. Similarly, we examined the functional connectivity between the mOFC and the VS in Self and Other conditions.

## Electronic supplementary material

Supplementary Information
