# Supplementary material for: Deciding for others as a neutral party recruits risk-neutral perspective-taking: Model-based behavioral and fMRI experiments
Source: Sci Rep. 2018 Aug 27;8:12857. doi: 10.1038/s41598-018-31308-6 (PMC6110713; doi:10.1038/s41598-018-31308-6)
Supplement: Supplementary file 1 — Supplementary Information [file 41598_2018_31308_MOESM1_ESM.pdf]

Deciding for others as a neutral party recruits risk-neutral perspective-taking: Model-based behavioral and fMRI experiments

Akitoshi Ogawa[1,2,3], Atsushi Ueshima[4], Keigo Inukai[5], Tatsuya Kameda[4*]

[1]*Department of Neurophysiology, Juntendo University School of Medicine, 2-1-1 Hongo, Bunkyo-ku, Tokyo 113-8421, Japan*

[2]*Brain Science Institute, Tamagawa University, 6-1-1 Tamagawagakuen, Machida, Tokyo 194-8610, Japan.*

[3]*Labratory for Symbolic Cognitive Development, RIKEN Center for Biosystems Dynamics Research, 2-1 Hirosawa, Wako, Saitama 351-0198, Japan.*

[4]*Department of Social Psychology, The University of Tokyo, 7-3-1 Hongo, Bunkyo-ku, Tokyo 113-0033, Japan.*

[5]*Faculty of Economics, Meiji Gakuin University, 1-2-37 Shirokanedai, Minato-ku, Tokyo 108-8636, Japan.*

*Direct correspondence to:

Tatsuya Kameda, Ph.D. (tkameda@l.u-tokyo.ac.jp)

Department of Social Psychology, The University of Tokyo

7-3-1 Hongo, Bunkyo-ku, Tokyo 113-0033, Japan

Tel: +81-3-5841-3870, Fax: +81-3-3815-6673

Table S1 | Thirty-six choice pairs used in the behavioral and fMRI experiments

Pair no.	Option	π_1	π_2	π_3	EV	Min
1	1	290	500	1010	600	290
	2	350	380	1070	600	350
2	1	150	780	870	600	150
	2	50	800	950	600	50
3	1	230	620	950	600	230
	2	200	320	1280	600	200
4	1	260	560	980	600	260
	2	160	220	1420	600	160
5	1	350	560	890	600	350
	2	330	810	960	700	330
6	1	360	540	900	600	360
	2	190	880	1030	700	190
7	1	330	600	870	600	330
	2	150	750	1200	700	150
8	1	330	600	870	600	330
	2	210	420	1470	700	210
9	1	400	640	760	600	400
	2	630	660	1110	800	630
10	1	470	500	830	600	470
	2	460	760	1180	800	460
11	1	440	560	800	600	440
	2	380	680	1340	800	380
12	1	440	560	800	600	440
	2	380	440	1580	800	380
13	1	470	680	1550	900	470
	2	240	1140	1320	900	240
14	1	290	1040	1370	900	290
	2	100	1150	1450	900	100
15	1	200	1220	1280	900	200
	2	210	660	1830	900	210
16	1	220	1180	1300	900	220
	2	40	730	1930	900	40
17	1	600	690	1410	900	600
	2	490	1120	1390	1000	490
18	1	390	1110	1200	900	390
	2	280	1240	1480	1000	280
19	1	480	930	1290	900	480
	2	130	1240	1630	1000	130

Pair no.	Option	π_1	π_2	π_3	EV	Min
20	1	480	930	1290	900	480
	2	390	420	2190	1000	390
21	1	610	940	1150	900	610
	2	730	1180	1390	1100	730
22	1	630	900	1170	900	630
	2	550	1210	1540	1100	550
23	1	710	740	1250	900	710
	2	410	1160	1730	1100	410
24	1	550	1060	1090	900	550
	2	10	1630	1660	1100	10
25	1	250	1660	1690	1200	250
	2	710	740	2150	1200	710
26	1	600	960	2040	1200	600
	2	580	640	2380	1200	580
27	1	690	780	2130	1200	690
	2	380	680	2540	1200	380
28	1	510	1140	1950	1200	510
	2	300	480	2820	1200	300
29	1	670	1180	1750	1200	670
	2	630	1470	1800	1300	630
30	1	640	1240	1720	1200	640
	2	270	1800	1830	1300	270
31	1	790	940	1870	1200	790
	2	590	770	2540	1300	590
32	1	720	1080	1800	1200	720
	2	510	540	2850	1300	510
33	1	910	1060	1630	1200	910
	2	1010	1340	1850	1400	1010
34	1	760	1360	1480	1200	760
	2	660	1620	1920	1400	660
35	1	870	1140	1590	1200	870
	2	610	1300	2290	1400	610
36	1	910	1060	1630	1200	910
	2	660	780	2760	1400	660

Note. The third through fifth columns indicate amounts of the three equiprobable (1/3) outcomes (in JPY). The sixth column indicates the expected value, and the seventh column indicates the minimum amount for each option. The order of the two options in each pair and the order of the 36 pairs were randomized across participants and the two tasks, and did not follow the order of entries in the table. Pearson's correlation between the two key task parameters (ΔEV and ΔMin) was not significant, $r_{\Delta EV, \Delta Min} = 0.06$, $P = 0.74$.

Table S2 | Estimated maximin-weight (α) of the Quasi-Maximin model[11], and fitness-comparison with the Expected Value model using Akaike information criterion (AIC). Standard errors of the mean in parentheses.

Experiment	Condition	α of Quasi-Maximin model	AIC of Quasi-Maximin model	AIC of Expected Value model
Behavioral (Mouselab)	Self	0.33 (0.03)	35.2 (1.3)	43.4 (1.3)
	Other	0.31 (0.03)	36.3 (1.2)	43.2 (1.3)
fMRI	Self	0.22 (0.05)	39.6 (1.5)	42.3 (1.8)
	Other	0.29 (0.07)	40.6 (1.5)	43.1 (1.7)

Note. AIC value was calculated using the following equation: $AIC = 2k - 2\ln(L)$, where k indicates the number of parameters and L indicates the likelihood.

Table S3 | Activation of Self vs. Other and Other vs. Self, found in whole brain analysis with a very liberal threshold (P-unc. < .01 for cluster identification and the cluster size threshold k = 60 voxels).

Contrast/Regions	MNI coordinates of the peak (mm) *x*	*y*	*z*	*Z*-score (peak)	P-unc. (cluster)	Number of voxels
Self > Other						
R Caudate	15	9	23	3.37	.141	74
L AI	–33	18	–10	3.33	.064	123
R MFG	27	18	53	3.32	.022	202
L Calcarine	–30	–66	8	3.01	.166	65
Other > Self						
L AG	57	–69	23	3.22	.114	73

L = left, R = right, AI = anterior insular, AG = angular gyrus, MFG = middle frontal gyrus.

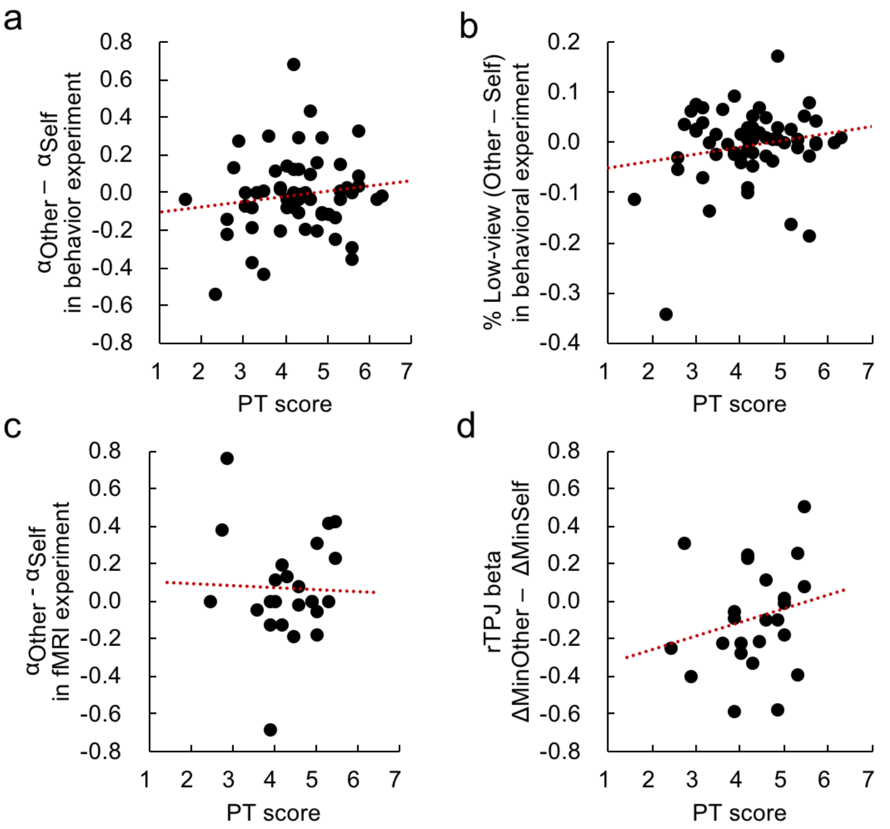

Fig. S1 | Correlations of participants' self-reported perspective taking (PT) scores of the IRI[14] with behaviors and brain activation. **a.** Correlation between PT scores and the difference between the maximin weights (α) in the Other and Self conditions ($r = .14$, $P = .29$), corresponding to Fig. 1d. **b.** Correlation between PT scores and the difference between the percentages of the low ("L") box view in the Other and Self conditions ($r = .19$, $P = .16$), corresponding to Fig. 1e. **c.** Correlation between PT scores and the difference between the maximin weights (α) in the Self and Other conditions in the fMRI experiment (robust correlation, $r = 0.01$, $P = .96$), corresponding to Fig. 2d. **d.** Correlation between PT scores and the difference between the modulatory effects of ΔMin on the rTPJ activity in the Other and Self conditions (robust correlation, $r = .20$, $P = .34$), corresponding to Fig. 3d. Lines indicate linear trends.

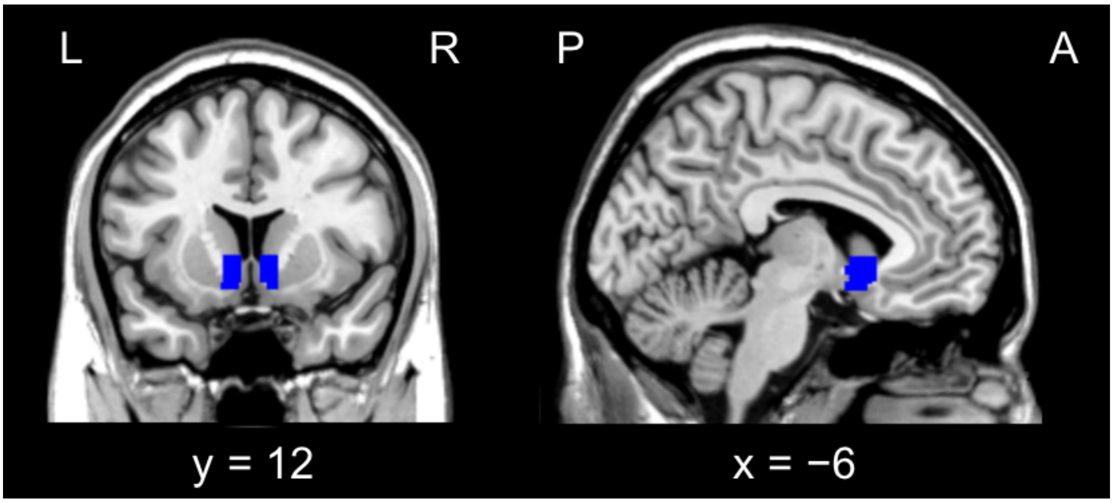

Fig. S2 | ROI definition of Ventral Striatum (VS) using MarsBaR[38]
